# A Case-Based Literature Review of RELA Associated Inflammatory Diseases

**DOI:** 10.1007/s10875-025-01968-x

**Published:** 2026-01-27

**Authors:** Nihal Karaçayır, Merve Yazol, Emine Nur Sunar Yayla, Pelin Esmeray Şenol, Çisem Yıldız, Nuran Belder, Merve Kutlar, Batuhan Küçükali, Büşra Acun, Deniz Gezgin Yıldırım, Sevcan A. Bakkaloğlu

**Affiliations:** 1 Department of Pediatric Rheumatology, Gazi University Faculty of Medicine, Ankara, Turkey; 2 Department of Pediatric Radiology, Gazi University Faculty of Medicine, Ankara, Turkey; 3 Clinic of Pediatric Rheumatology, Ankara Etlik City Hospital, Ankara, Turkey; 4 Clinic of Pediatric Rheumatology, Mersin City Hospital, Mersin, Turkey

**Keywords:** RELA, Ulcer, Behcet’s disease, Hereditary autoinflammatory disease, NF-κB, Interferonopathy

## Abstract

Behçet’s disease (BD) is a chronic inflammatory disorder characterized by recurrent oral aphthous ulcers, genital ulcers, skin lesions, and uveitis. Recent genetic studies have identified monogenic diseases with phenotypes resembling BD, including RELA*-*associated inflammatory disease (RAID), *Haploinsufficiency of A20* (HA20), and otulipenia. The *RelA* gene encodes the RELA protein, which is involved in the nuclear factor kappa B (NF-κB) signaling pathway that regulates the transcription of genes associated with cell survival, apoptosis, and immune responses. In RAID, dysfunction of the NF-κB pathway leads to reduced cell survival and symptoms of BD, such as recurrent fever, chronic mucocutaneous ulceration, arthralgia, and colitis. Herein, we report a pediatric patient who presented with recurrent, severe oral and genital ulcers from the age of five years and was diagnosed with RAID following a documented *RelA* gene mutation. The patient responded to a combination of corticosteroids, colchicine and methotrexate. RAID should be considered in the differential diagnosis of patients with early onset recurrent fever and mucosal ulcerations.

## Introduction

Behçet’s disease (BD) is a chronic inflammatory disorder first described in 1937 by Hulusi Behçet, which presents with recurrent aphthous stomatitis, genital ulcers, erythema nodosum, and uveitis. The etiology of BD is thought to involve both environmental and genetic factors. From a genetic perspective, the presence of HLA-B5101 is the strongest genetic risk factor, associated with a six-fold increase in the risk of developing BD. Large genome-wide association studies have further demonstrated an association with BD susceptibility genes such as *IL10*, *IL23R/IL12RB2*, *STAT4*, and *ERAP1* [1]. Although aphthous ulcers and inflammation of the oropharyngeal mucosa are hallmarks of Behçet’s disease, these features are also observed in other disorders, including PFAPA syndrome and Behçet-like genetic diseases. While PFAPA syndrome shares common genetic risk loci with Behçet’s disease in the *IL12A*, *STAT4*, *IL10*, and *CCR1–CCR3* gene regions, some monogenic diseases present with a BD-like clinical phenotype but are caused by distinct genetic mutations [2, 3]. 

BD shares common pathogenic mechanisms with both autoimmune and autoinflammatory diseases [2]. Autoinflammatory diseases are genetic disorders typically characterized by early onset systemic inflammatory attacks. The genetic etiology involves abnormalities in molecules such as inflammasomes, cytokine receptors and inhibitors, metabolic enzymes, and/or proteasome complexes [4]. Monogenic autoinflammatory diseases are caused by inborn genetic errors and can be divided into subgroups according to the molecular mechanisms: inflammasomopathies, interferonopathies, protein misfolding, endogenous antagonist deficiencies and NFκBopathies [5, 6]. Dysregulation of the NF-κβ pathway is a common finding in disorders with BD or BD-like phenotypes. This pathway is crucial for maintaining self-tolerance and regulating immune and inflammatory responses by controlling inflammatory, pro-apoptotic, and anti-apoptotic gene expression. It regulates numerous cellular processes, including cytokine production, cell proliferation, cell survival, and host immunity. Mutations in this pathway result in inborn errors of immunity, which can manifest as immune deficiency and autoinflammation [2, 7, 8]. A heterozygous loss-of-function mutation in *RelA* on chromosome 11q13.1, which encodes the RELA (p65) protein involved in this pathway, leads to the development of an autosomal dominant disorder, known as RELA-associated inflammatory disease (RAID). This mutation leads to dysfunction in the pathway, with decreased anti-apoptotic gene expression, causing increased apoptosis and phenotypic features of BD-like disease, including fever, skin rash, arthritis, arthralgia, myalgia, oral and genital ulcers, uveitis, immunodeficiency, and pathergy test positivity [4, 9–11]. 

Herein, we report a pediatric patient who presented with clinical manifestations of BD and was diagnosed with RAID, with a documented heterozygous *RelA gene* mutation. We also reviewed and summarized the current literature on the symptoms, laboratory findings, genetics, treatments, and treatment responses of patients with RAID.

## Case Presentation

A fourteen-year-old female patient presented with recurrent fever, aphthous lesions in the oral cavity, and arthralgia, which had been present since the age of five. On admission, the patient had no uveitis or genital ulcers. The patient had 3 healthy younger siblings. There was no consanguineous marriage between the parents and no family history of autoimmune, autoinflammatory, or immunodeficiency disorders; however, her mother had oral ulcers only during her teenage years. On physical examination, the patient had an aphthous lesion on the inner surface of the upper lip, approximately 0.5 cm in diameter. The pathergy test result was negative. Laboratory work-up, including complete blood count, biochemistry, urinalysis, complement components C3 and C4, and immunoglobulin levels, were within normal ranges. The erythrocyte sedimentation rate (ESR) was normal (6 mm/h), and the C-reactive protein (CRP) level was slightly elevated (6.75 mg/L, normal range < 5 mg/L). HLA-B5101 was negative, antinuclear antibody (ANA) was 3 + with granular staining, and the ENA profile was negative. Colchicine was initiated, leading to a reduction in the frequency and severity of oral ulcers. At the age of 12, the patient presented with a 2 × 1 cm ulcer on the right labium majora. The genital ulcer progressed to an abscess during the follow-up period. The white blood cell count was elevated, with a predominance of neutrophils. ESR was 34 mm/h, and CRP was 265 mg/L. ANA was positive at 2 + with a granular staining pattern, whereas the ENA profile, ANCA, and viral serologies remained negative. Blood cultures and swabs of the genital abscess were negative. Magnetic resonance imaging of the perineum revealed a collection with peripheral enhancement in the vulvar region, adjacent to the distal parts of the vagina and the urethra. The collection showed diffusion restriction, consistent with abscess. The results of upper gastrointestinal endoscopy and colonoscopy were normal. Based on the clinical presentation, the patient was diagnosed with BD. In addition to local skin care, we added glucocorticoid (GC, 1 mg/kg/day) and azathioprine (AZA, 2 mg/kg/day) to colchicine (COL), which led to the resolution of the genital abscess. The GC was tapered and stopped within two months. Up until the age of 16, the patient had recurrent genital ulcers, which were relieved with systemic GC therapy. We switched AZA to methotrexate (MTX) due to the severe and resistant course of the ulcerative lesions, ongoing fever, and the frequent need for steroids. The patient responded well to the combination of colchicine and MTX and remained in remission at one-year follow-up.

Due to the early age of onset and severe phenotype, whole exome sequencing (WES) was performed to investigate the monogenic causes of BD. We detected a heterozygous mutation causing a frameshift in *RelA* (c.1303dup; p. Thr435fs), leading to the final diagnosis of RAID. The mutation had not been previously reported but was classified as likely pathogenic according to the 2015 American College of Medical Genetics and Genomics (ACMG) criteria [12]. WES performed on the patient’s mother, who had oral ulcers during her teenage years, did not reveal any *RelA* mutation. The patient’s father declined to undergo further genetic testing.

### Search Strategy

**Fig. 1:**
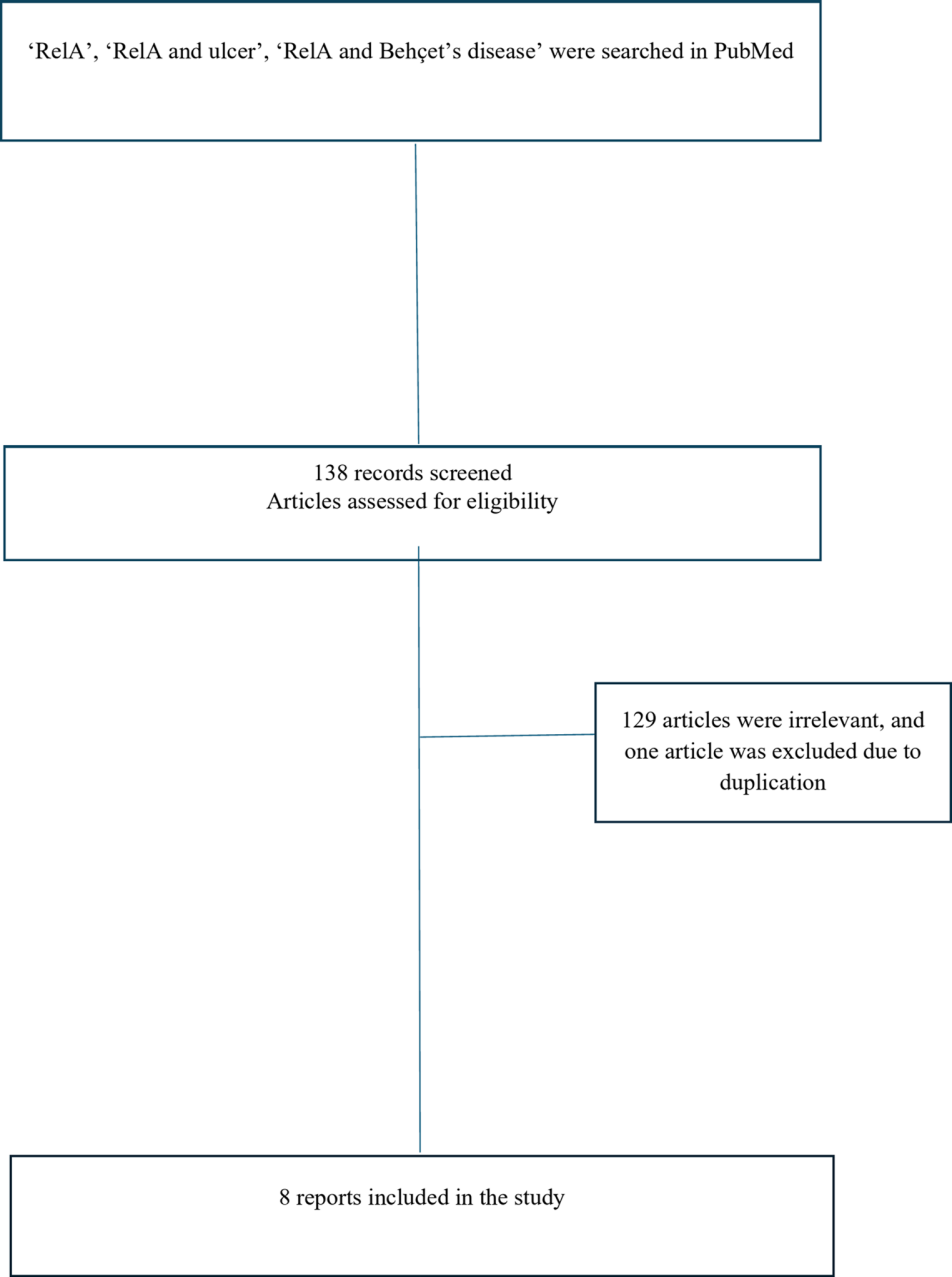
Literature search terms and strategy in PubMed

We searched PubMed Medline using the keywords’ RELA’, ‘RELA and ulcer’, and ‘RELA and Behçet’s disease’ and reviewed the current English literature from inception to September 2024 regarding clinical cases. A total of 138 articles were identified. Studies on experimental treatments and animal models were excluded. The references of the existing articles were also reviewed. A total of eight studies, encompassing 42 clinical cases, were identified. The search strategy is illustrated in Fig. 1. Age, sex, laboratory and genetic results, radiological and clinical findings, and treatment modalities were recorded for all patients. Clinical findings are summarized in Table 1. The detailed findings of the patients are presented in Table 2.

**Table 1:** Clinical manifestations of patients with RAID

Clinical Features/Organ system involvement	Number (*n* = 43)	%
Oral ulcers	26	60
Genital ulcers	14	32
Rash	14	32
Recurrent fever	15	34
Gastrointestinal	9	20
Musculoskeletal	13	30
Central nervous	6	13
Ocular	6	13
* Conjunctivitis *	4	
* Episcleritis*	1	
* Recurrent optic neuritis*	1	
Cardiovascular	2	5
Renal	2	5

**Table 2:** Literature review and summary of patients carrying *RelA* mutations

Article	Patient no	Sex/ethnicity	Symptom onset	Clinical symptoms and pathological examination findings	Laboratory findings	Pathological findings	Recurrent infection	Autoantibody	HLAB51	Previous diagnose	Genetic mutation/ ACMG classification*/ClinVar ID	Treatment
Frederiksen et al. 2016 [13]	**P1**	M/NA	7 days	Respiration stopped suddenly at home	NA	High bone mass	NA	NA	NA	Unexplained neonatal death	*c.1534_1535delinsAG* (p. Asp512Ser) *Heterozygous*,* missense mutation/VUS/NA*	None
Badran et.al. 2017 [9]	**P1**	F/NA	3 yr	Abdominal pain, vomiting, fever and oral ulcers	Leucocytosis and elevated APR	Acute ileitis	None	Negative	NA	Chronic mucocutaneous ulceration	*c.559 + 1G >A* *Heterozygous splice site mutation/Pathogenic*/*617,487*	AZA, COL, Anakinra (non-responsive) GC, IFX + MTX (responsive)
	**P2**	F/NA	2 yr	Oral and genital ulcers	NA	None	NA	NA	NA	Same disease	Same mutation	AZA, COL (non-responsive) GC (responsive)
	**P3**	M/NA	2 yr	Oral ulcers, diarrhea	NA	None	NA	NA	NA	Same disease	Same mutation	NA
	**P4**	F/NA	8 yr	Oral ulcers	NA	None	NA	NA	NA	Same disease	Same mutation	NA
Comrie et al. 2018 [14]	**P1**	M/NA	5 yr	Splenomegaly, LAP	Thrombocytopenia, anaemia, neutropenia	Aseptic meningitis	NA	ANA negative ENA Negative	NA	ALPS	***c.736 C >T (1)*** (p. Arg246*) *Heterozygous nonsense mutation/* *Pathogenic/617,486*	Splenectomy, Eltrombopag, (partial response) GC, MMF, IVIG, RTX (Responsive)
Bernabei et al. 2020 [15]	**P1**	F/Algerian	1,5 yr	Malar rash, oral ulcers, arthralgia, alopecia, fever, cutaneous rash, skin ulceration	Low C4	Vasculitis, pubertal delay	Chronic suppurative otitis media	ANA, anti-ds DNA, anti-SSa, and anti-SSb antibodies ACL, anti-B2GP1 RF positive	NA	SLE	*c.256 C >A* (p.H86N) *Heterozygous missense mutation* */Likely Pathogenic*/*NA*	MMF, RTX (non-responsive) GC (responsive)
	**P2**	F/Algerian	33 yr	Polyarthritis, oral ulcers	Low C4	Pericarditis, class IV lupus-nephritis	NA	ANA, anti-dsDNA positive	NA	SLE	Same mutation	GC, MMF, CYC (responsive)
	**P3**	M/French	9 yr	Malar rash, oral ulcers, arthralgia, rash, skin ulceration	NA	Pericarditis, class IV lupus nephritis	NA	ANA, anti-ds DNA, anti-SSA, anti-SSB	NA	SLE	***c.985 C >T (2)*** (p.R329X) *Heterozygous nonsense mutation* *Pathogenic/* *1,075,392*	HCQ, GC, MMF (responsive)
Adeeb et.al. 2021 [10]	**P1**	M/Irish	15 yr	Recurrent oral ulcers	NA	None	NA	NA	Negative	NA	*c.1459del* (p. His487ThrfsTer7) *Heterozygous frameshift mutation/Pathogenic/* *2,446,403*	None required
	**P2**	F/Irish	10 yr	Recurrent oral ulcers, pustulosis and acneiform rash	NA	None	NA	ANA negative	Negative	BD	Same mutation	ETC (responsive)
	**P3**	F/Irish	15	Recurrent oral and genital ulcers, acneiform rash	NA	None	NA	ANA negative	Negative	BD	Same mutation	ETC (responsive)
	**P4**	F/Irish	22	Recurrent optic neuritis	NA	None	NA	AQP-4 antibody	Negative	NMO	Same mutation	RTX (responsive)
	**P5**	F/Irish	10	Recurrent oral ulcers	NA	None	NA	ANA negative	NA	NA	Same mutation	None required
Lecerf et al. 2022 [7]	**P1**	F/Eurasian	Infant	Recurrent oral and genital ulcers, raised intraocular pressure and thinning of the optic nerve	Elevated APR	Congenital heart disease, environmental allergies	NA	Negative	NA	BD	*c.1044dupC* (p. Tyr349LeufsTer13*)* *Heterozygous frameshift mutation* */Likely Pathogenic/NA*	AZA (non- responsive) GC, sucralfate, COL (responsive)
	**P2**	M/Eurasian	Infant	Recurrent fever, oral and genital ulcers, episcleritis, leg pain, urticarial rash, diarrhea, poor weight gain, erythema nodosum	Elevated APR	None	NA	Negative	Negative	BD	Same mutation	GC, COL (partial response)
	**P3**	F/Eurasian	NA	NA	NA	NA	NA	NA	NA	BD	Same mutation	NA
	**P4**	F/Eurasian	12 yr	Recurrent oral and genital ulcers	NA	None	NA	NA	NA	Crohn disease BD	Same mutation	GC, HCQ, COL AZA (non-responsive) IFX, ADA (responsive, side effect) Apremilast (responsive)
	**P5**	M/Eurasian	1 month	Recurrent fever, oral and anogenital ulcers, conjunctivitis, urticarial like rashes, poor weight gain, cervical LAP	Elevated APR thrombocytosis and anaemia	None	None	NA	Negative	BD	Same mutation	GC, COL, AZA (partial response) Anakinra, canakinumab (responsive)
	**P6**	F/Eurasian	NA	NA	NA	NA	NA	NA	NA	Crohn disease BD	Same mutation	NA
	**P7**	F/Eurasian	NA	NA	NA	NA	NA	NA	NA	Crohn disease BD	Same mutation	NA
An et al. 2023 [16]	**Family 1** **P1**	F/Canadian	29	Recurrent oral and genital ulcers, rash, arthralgias, myalgia, night sweats	Elevated APR	None	NA	ANA negative	NA	BD	*c.1153 C >T* (p. Gln385Ter) *Heterozygous nonsense mutation/Pathogenic/* *1,452,423*	MTX (non- responsive) COL, ETC, GC (responsive)
	^**P2**^	M/Canadian	9	Recurrent oral ulcers	NA	None	NA	ANA negative	NA	NA	Same mutation	COL (responsive)
	**P3**	F/Canadian	8	Recurrent oral and genital ulcers, fever, headache, brain fog	NA	None	NA	NA	NA	Neuro-Behcet’s syndrome	Same mutation	GC, COL, HCQ (responsive)
	**P4**	F/Canadian	15	Recurrent oral and genital ulcers, fatigue	NA	None	NA	NA	NA	NA	Same mutation	GC (responsive)
	**Family 2** **P5**	M/Brazilian	Early life	Rash	NA	None	NA	NA	NA	NA	*c.1311dup* (p. E438Rfs*9) *Heterozygous Frameshift mutation* */Likely Pathogenic/NA*	None required
	**P6**	F/Brazilian	2 yr	Recurrent oral and genital ulcers, fatigue, arthralgia, periodontitis, rash, headache, atopic dermatitis, skin ulceration, folliculitis	Elevated APR	None	NA	ANA negative		BD	Same mutation	GC, MTX, COL, THAL (non- responsive) IFX, ADA (responsive)
	**P7**	F/Brazilian	3 yr	Recurrent oral and genital ulcers, fever, arthralgias, rash, atopic dermatitis, folliculitis, skin ulcerations	NA	None	NA	ANA positive	NA	BD	Same mutation	GC, COL, MTX (non-responsive) IFX (responsive)
	**P8**	F/Brazilian	40 days	Recurrent oral, perianal, and genital ulcers, fever, pustular rash, folliculitis, headache	Elevated APR	Esophagitis and colitis, multifocal white matter lesions, renal calculi	NICU for pneumonia, sepsis, cellulitis, recurrent otitis media, pharyngitis, appendicitis	ANA positive	NA	BD	Same mutation	GC, COL, MTX, AZA (non- responsive) ADA (responsive)
	**P9**	M/Brazilian	5 yr	Recurrent oral, genital, and anal ulcers, rash, headache, folliculitis, skin ulceration	Elevated APR	None	NA	ANA negative	NA	BD	Same mutation	GC, COL, MTX, AZA (non- responsive) ADA (responsive)
	**Family 3** **P10**	M/European	9 yr	Recurrent fever, bloody diarrhea, arthralgia, peritonitis	Elevated APR	Leukocytoclastic vasculitis, mesenteric lymphadenitis	NA	ANA negative	NA	NA	***c.985 C >T (2)*** (p.R329*) *Heterozygous nonsense mutation; Pathogenic/1,075,392*	COL, ANA (non- responsive) ETC (responsive)
	**P11**	F/European	10 yr	Recurrent fever, knee swelling, conjunctivitis, rash, tendinitis, sicca, erythema nodosum	Elevated APR	None	NA	NA	NA	Sjogren’s syndrome and SLE	Same mutation	GOL, ADA (NA)
	**P12**	F/European	10 yr	Abdominal symptoms	NA	Inflammation in colon	NA	NA	NA	NA	Same mutation	NA
	**Family 4** **P13**	M/European	Birth	Recurrent fever, oral ulcers, vesicular rash, diarrhea, hypotonia, poor weight gain, skin ulcerations	Elevated APR	None	Infections with respiratory syncytial virus, croup, and otitis media	NA	NA	NA	***c.736 C >T (1)*** p.R246* *Heterozygous* *nonsense mutation/* *Pathogenic/* *617,486*	GC, ETC (responsive)
	**P14**	M/European	2 yr	Abdominal pain, oral ulcer	NA	None	NA	NA	NA	NA	Same mutation	None required
	**P15**	F/European	26 yr	Conjunctivitis	NA	None	NA	NA	NA	NA	Same mutation	NA
Moriya et al. 2023 [17]	**P1**	M/Japanese	1 month	Recurrent fever, tonsillitis, abdominal pain, refractory diarrhea, cervical LAP	NA	Ulcers in the colon, scoliosis	NA	NA	NA	Intestinal BD	*c.1165 C >T* p.Q389* *Heterozygous nonsense mutation*/*Likely Pathogenic/NA*	IFX (responsive)
	**P2**	F/Japanese	Childhood	Recurrent aphthous stomatitis, fever, arthralgia	NA	None	NA	NA	NA	JIA	Same mutation	NA
	**P3**	M/ Japanese	Newborn	Perianal abscess, diarrhea	Neutropenia, thrombocytopenia	None	NA	Antineutrophil antibody positive	NA	Crohn’s disease, ITP, autoimmune neutropenia	***c.985 C >T (2)*** p.R329* Heterozygous *nonsense mutation*; *Pathogenic/* *1,075,392*	RTX, tacrolimus (non-responsive) Hematopoietic stem cell transplantation (responsive)
	P4	M/Japanese	2 yr	Recurrent fever, painful subcutaneous nodules, growth failure, myalgia, stomatitis	Elevated IgG and IgD.	None	NA	ANA positive	NA	NA	*c.1416dup* p. E473Rfs*18 *Heterozygous frameshift mutation/* *Pathogenic/* *1,453,182*	Cimetidine (non-responsive)
	**P5**	F/Japanese	6 months	Periodic fever, growth failure, abdominal pain	NA	Ulcers in the colon, recurrent pancreatitis, aortic regurgitation, hypothyroidism	NA	ANA negative	NA	Autoinflammatory disease of unknown etiology, IBD	*c.1034-1G >A Heterozygous splicing site mutation/* *Likely Pathogenic*/*NA*	NSAIDs, Tocilizumab, ADA (responsive)
	**P6**	M/Moroccan	18 months	Bullous rash, conjunctivitis, facial edema, macrocheilitis, diffuse xerosis, subcutaneous nodules, LAP	Elevated APR	Short stature, delayed puberty	Recurrent bacterial and mycotic infections	NA	NA	NA	*c.1047T >A* p.Y349* *Heterozygous mutation/Likely* *Pathogenic/NA*	GC (partial response)
Our study	**P1**	F/Turkish	5 yr	Recurrent fever, oral and genital ulcers, vulvar abscess, arthralgia	Elevated APR Leucocytosis	None	None	ANA positive	Negative	BD	*c.1303dup* p. Thr435Fs *Heterozygous* *Frameshift mutation* */Likely pathogenic/NA*	AZA (partial response) Colchicine, MTX, GC (responsive)

*ADA *Adalimumab, *ALPS *Autoimmune Lymphoproliferative Syndrome, *ANA *Antinuclear antibodies, *anti-dsDNA, *Anti–double-stranded DNA, *APR, *Acute phase reactants, *AZA *Azathioprine, *BD * Behcet’s disease, *COL* Colchicine, *CYC* Cyclophosphamide, *ETC* Etanercept, *GC* Glucocorticoid, *HCQ* Hydroxychloroquine, *IFX* Infliximab, *IBD* Inflammatory bowel disease, *ITP* Immune thrombocytopenic purpura, *IVIG *Intravenous immunoglobulin, *JIA* Juvenile idiopathic arthritis, *LAP* lymphadenopathy, *MTX* Methotrexate, *MMF *Mycophenolate mofetil, *NMO* Neuromyelitis Optica, *NSAID* Non-steroid anti-inflammatory drug, *RF* Rheumatoid factor, *RTX* Rituximab, *SLE* Systemic lupus erythematosus, *THAL* Thalidomide

*ACMG: American College of Medical Genetics and Genomics: We assessed part of the genes’ pathogenicity from the Clinvar database, and for those not available there, we evaluated them ourselves based on 2015 ACMG criteria [12]

(1) and (2): The same genes were highlighted in bold with the same numbers in parentheses.

## Results

### Demographic Data, Clinical and Laboratory Characteristics

Data from 43 patients reported in the literature, including our own case, were analyzed. Among them, 26 were female, with a predominance of Eurasian origin (*n* = 7). Other reported ethnicities included six of European origin; five each of Japanese, Irish Caucasian, and Brazilian origin; four Canadian; two Algerian; and one each of French Caucasian, Moroccan, and Turkish origin. The mean age at diagnosis was 5,97 years (range, 0–33 years). Of these patients, 23% were diagnosed before the age of one year. Among them, four were index cases, and six were diagnosed based on a positive family history. Only three patients were diagnosed in adulthood.

The patients’ diagnoses prior to genetic testing included BD (*n* = 17), chronic mucocutaneous ulceration (*n* = 4), systemic lupus erythematosus (SLE) (*n* = 4), juvenile idiopathic arthritis (*n* = 1), inflammatory bowel disease (IBD) (*n* = 3), autoimmune lymphoproliferative syndrome (ALPS) (*n* = 1), Crohn disease (CD) (*n* = 4), immune thrombocytopenic purpura (ITP) (*n* = 1), autoimmune neutropenia (*n* = 1), and neuromyelitis optica (*n* = 1). BD was the most common diagnosis. Patients presented with BD-like clinical features including, genital ulcers, acneiform rash, oral ulcers, erythema nodosum, refractory diarrhea, and joint inflammation [7, 10, 16]. One patient had recurrent oral and genital ulcers, fever, headache, and brain fog, and was diagnosed with neuro-Behçet disease [16]. The other patient had recurrent fever and diarrhea starting from one month of age and was diagnosed with intestinal BD [17].

The most common symptoms were ulcers affecting the oral, gastrointestinal, and genital mucosa. Oral ulcers were observed in 60% of the patients (26 cases), and among them, 14 had both oral and genital ulcerations. A vulvar abscess was observed in one patient, and a perianal abscess in another. Abdominal symptoms (abdominal pain, diarrhea and vomiting), were observed in 9 patients, six of whom had diarrhea. Recurrent fever was observed in 34% of the patients. Rash was present in 32% of patients and included acneiform, pustular, urticarial, vesiculobullous, erythema nodosum, folliculitis, ulcers, vasculitis, atopic dermatitis, and malar rash. Skin ulcers were observed in five patients. Musculoskeletal symptoms, including arthritis, arthralgia, and myalgia, were observed in 30% of the patients. Central nervous system involvement was observed in six patients: four experienced headaches, one had brain fog, and one presented with aseptic meningitis. Pericarditis and lupus nephritis were identified in two patients with SLE. Other findings include fatigue, growth retardation, delayed puberty, hypothyroidism, hypotonia, night sweats, kidney stones, periodontitis, recurrent tonsillitis, pancreatitis, and stomatitis. Only three patients had recurrent infections. The clinical findings are summarized in Table 1.

Fourteen different gene mutations were identified in the literature. Of these, one was reported in three separate publications, and another was reported in two. The pathogenicity of all identified genes was assessed, but only some were listed in the ClinVar database. For variants not available in ClinVar, pathogenicity was assessed based on the 2015 ACMG guidelines [12]. All mutations were classified as either pathogenic or likely pathogenic.

Acute-phase responses were elevated in one-third of patients. Anemia and neutropenia were each observed in two patients, while lymphopenia was present in one. Low C4 levels were observed in two of the patients. ANA was tested in 19 patients, with positive results observed in seven. Three of these patients were diagnosed with SLE. HLA B-5101 was tested in six patients, and all were negative.

##  Discussion

In this report, we present a pediatric patient with early onset recurrent fever, oral and genital ulcers, and vaginal abscesses who was initially diagnosed with BD and later confirmed to have RAID by genetic analysis. Owing to the rarity of this disease, we conducted a literature review on RAID, as shown in Fig. 1.

**Fig. 2:**
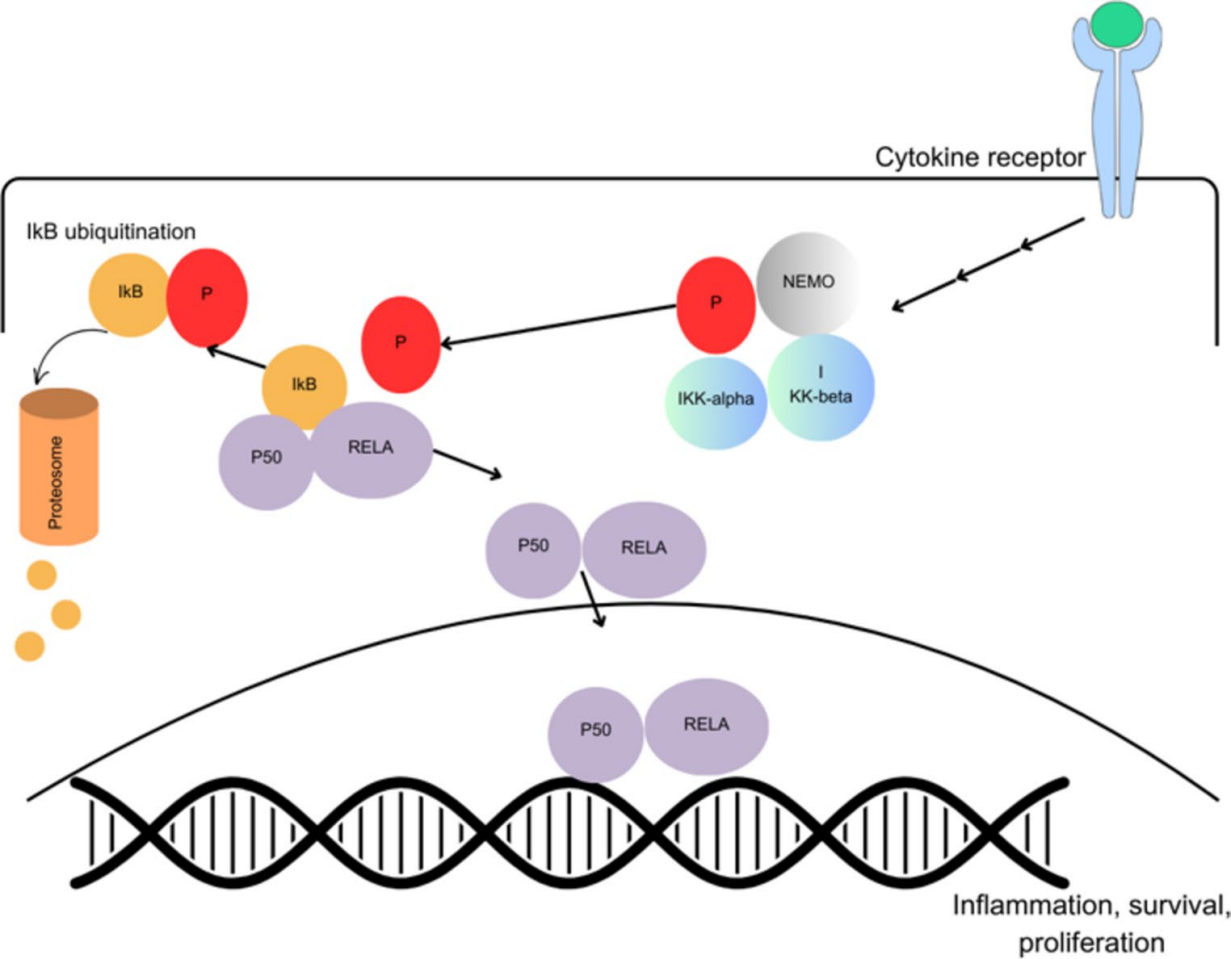
RELA protein function in the nuclear factor kappa B activation pathways. In response to cytokines such as TNF-α, IL-1β and IL-6 the IκB protein, which is bound to the p50/RelA heterodimer, is phosphorylated by the IκB kinase (IKK) complex (IKKα, IKKβ, and NEMO). Phosphorylated IκB is then targeted for ubiquitination and degradation by the proteasome complex. The released heterodimer translocates to the nucleus, binds to the promoter regions of immune-related genes, and initiates their expression, which is essential for cell survival, inflammation, and immunity. Abbreviations: IkB: NF- κB inhibitor; IKK: IκB kinase; NEMO: NF-κB essential modulator; TNF-alpha: Tumor necrosis factor alpha. Figure 2 was prepared using Canva graphics program

RAID is an autosomal dominant disorder caused by heterozygous mutations in the *RelA* gene on chromosome 11q13.1, which encodes RELA (p65) protein. RELA is one of the five transcription factors that mediate the expression of NF-κB pathway genes. The other four are p50, p52, c-Rel, and RelB. These subunits form various homo- and heterodimers. The most abundant heterodimers are p50 and p65 (RELA). This heterodimer is found in the cytoplasm and is bound to an inhibitory protein known as NF-κB inhibitor (IκB). In response to various stimuli, such as TNF-α and IL-1, IκB is phosphorylated by the IκB kinase complex (IKK), which is subsequently ubiquitinated and degraded by proteasomes. Upon dissociation from IκB, the heterodimer translocates to the nucleus, binds to the promoter regions of immune-related genes, and initiates their expression, thereby regulating cell survival, inflammation, and host immunity [9, 11]. The NF-κB pathway is illustrated in Fig. 2.

TNF-α is a proinflammatory cytokine that plays a significant role in the regulation of inflammatory responses, cell cycle proliferation, and apoptosis. It activates divergent pathways, promoting caspase-8-mediated apoptosis and NF-κB-dependent cell survival [18]. Badran and colleagues [9] demonstrated that TNF-α is a key factor in increasing apoptosis in epithelial and stromal cells in the context of *RelA* mutation. They identified a splice-site mutation in *RelA*, causing an early stop codon, through WES analysis conducted on a mother with chronic mucosal ulcerations and her three effected children. This mutation resulted in a 50% decrease in the RELA protein expression. The fibroblasts of patients exhibited impaired NF-κB activation, defective expression of NF-κB–dependent antiapoptotic genes, and increased apoptosis in response to TNF-α. The authors identified that *RelA* mutations lead to dysregulation of the NF-κB signalling pathway, resulting in increased TNF-mediated cytotoxicity and apoptosis, ultimately suggesting that this may contribute to the development of mucocutaneous ulcers. 

Four patients were diagnosed with SLE [15, 16] and presented with malar rash, oral ulcers, sicca syndrome, leukocytoclastic vasculitis, alopecia, fever, arthritis, pericarditis, and lupus nephritis. SLE is characterized by the increased production of autoantibodies and inflammatory cytokines, such as type-1 interferons (IFNs). IFN-α supports B cell differentiation and immunoglobulin class switching to generate potentially pathogenic autoantibodies, leading to SLE [19]. In some *RelA* mutations, a reduction in RELA protein levels leads to RELA haploinsufficiency [9], while in others, mutant proteins interact with normal RELA protein, exerting a dominant-negative (DN) effect that alters gene expression in the NF-κB pathway and increases IFN levels [15, 17]. Bernabei et al. [15] demonstrated the overproduction of type-1 IFNs in three SLE patients with DN- RELA mutations. They demonstrated that co-expression of mutant and wild-type RELA strongly activated gene expression controlled by IFNα-consensus sequences, leading to the overproduction of type-1 IFNs. All patients tested positive for ANA and anti-double-stranded DNA. As mucosal ulceration is one of the criteria for SLE [20], autoantibodies were assessed in our patient to exclude SLE. The ANA titer was 3+; however, the patient did not meet the classification criteria for SLE.

In line with Bernabei’s study [15], Moriya et al. [17] demonstrated that some mutant RELA proteins have a DN effect by forming heterodimers with wild-type RELA and altering its function. They reported that patients with *RelA* DN mutations had more severe clinical symptoms than those with *RelA* haploinsufficiency mutations. The authors also identified elevated IFN levels in the sera of patients, similar to Bernabei’s study [15]. Based on the clinical and cellular characteristics of type I interferonopathy observed in patients with *RelA* DN mutations, Janus kinase (JAK) inhibitors have been proposed as a potential treatment option for cases unresponsive to anti-TNF or other therapies [17]. 

RAID can manifest differently in patients with the same mutations. Adeeb et al. [10] reported five patients with the same *RelA* mutation (c.1459del). Two were diagnosed with BD, two had only recurrent oral ulcers, and one had no BD-like symptoms but tested positive for AQP-4 antibodies and was diagnosed with neuromyelitis optica. In two different studies, one patient [16] had recurrent fever, oral ulcers, diarrhea, and rash since birth, while another patient [14], who shared the same mutation (c.736 C >T), was diagnosed with ALPS at the age of five years due to pancytopenia, splenomegaly, and lymphadenopathy. In Moriya’s study [17], one of the patients had a perianal abscess and diarrhea since the age of one month, was diagnosed with CD and ITP, and carried the same mutation (c.985 C >T) as the patient [16] who presented with recurrent fever, arthralgia, and bloody diarrhea, starting at the age of nine. In another study, Bernabei et al.[15] described a patient with the same mutation (c.985 C >T) who presented with oral and skin ulcers, arthralgia, lupus nephritis, and pericarditis. The same mutations in a gene can lead to diverse clinical phenotypes, depending on factors such as an individual’s genetic background, as well as epigenetic and environmental factors.

In addition to autoinflammatory and autoimmune diseases, *RelA* mutations have been associated with hyperostosis. In 2016, Frederiksen et al. [13] reported the first mutation of the NF-κB complex in humans, which was observed in an infant with unexplained sudden death. Postmortem examinations revealed radiological and histopathological findings consistent with high bone mass (HBM) in the infant. Screening for known genes associated with HBM yielded negative results. WES revealed a heterozygous mutation in *RelA* (c.1534_1535delinsAG). They showed a reduction in NF-κB activity in the fibroblasts of patients. NF-κB activation leads to increased osteoclast survival and decreased osteoblast maturation and function [21]. In cases of RELA deficiency, a decrease in NF-κB function could reduce osteoclast survival, ultimately resulting in reduced bone resorption and increased bone formation [13]. Hyperostosis has not been reported in other patients in the literature. This may be explained by the fact that different mutations within the same gene can result in distinct clinical phenotypes, depending on factors such as the type of mutation (e.g., gain- or loss-of-function), the specific functional domain affected, and the cellular context of the target tissue (e.g., immune cells vs. bone cells).

Evidence-based treatment recommendations for RAID are currently unavailable. In the literature, different presentations have been treated using various single or combination therapies (Table 2). Most patients with acute symptoms respond well to GC therapy. For chronic mucocutaneous ulcers or a preliminary diagnosis of BD anti-TNF therapies were effective, while AZA, MTX and COL were partially effective in some patients [7, 9, 10]. One patient diagnosed with CD [7] and BD did not respond to treatment with GC, COL, or AZA. She experienced severe complications with anti-TNF therapies but responded well to apremilast. RAID diagnosis did not affect our patient’s treatment. Her symptoms improved with MTX and COL treatment. However, if frequent relapses occur in the future, anti-TNF therapy will be considered.

## Conclusion

The most common clinical findings observed in patients with RAID are recurrent oral and genital ulcers, fever, and rash. Elevated acute-phase responses were observed in one-third of patients. Genetic testing for NFκBopathies, such as RAID, HA20, or Otulipenia, should be considered in patients with early-onset, recurrent oral and anogenital ulcers and/or abscesses, recurrent fever, elevated acute-phase reactants, and a positive family history of BD-like symptoms. If no mutations are detected, WES should be performed to identify novel mutations in other NF-κB pathway genes.

### Limitations

The main limitation of this study is the lack of functional validation for the new mutation presented here. However, based on in silico predictions from current databases and information from patients with similar clinical presentations, we believe that this mutation explains our patient’s clinical condition.

## Data Availability

Data sharing is not applicable; no new data were generated, or the article describes an entirely case report.
